# Adherence to Antiretroviral Therapy: Merging the Clinical and Social Course of AIDS

**DOI:** 10.1371/journal.pmed.0020338

**Published:** 2005-10-04

**Authors:** Arachu Castro

## Abstract

Compliance with HIV treatment is affected by many issues and social factors are as important as biological ones.

The survival of people diagnosed with HIV/AIDS dramatically improves with access to highly active antiretroviral therapy (HAART). Such therapy employs a combination of antiretroviral agents—protease inhibitors (PIs), nucleoside reverse transcriptase inhibitors, non-nucleoside reverse transcriptase inhibitors, nucleotide reverse transcriptase inhibitors, and fusion inhibitors—to suppress viral replication, and, thus, reduces the likelihood of developing HIV mutations that could lead to the development of drug-resistant viral strains. HAART also prevents further viral destruction of the cellular immune system, thereby, allowing for increases in the level of CD4+ cells, which improves the immunologic response to opportunistic infections. [Fig pmed-0020338-g001]

**Figure pmed-0020338-g001:**
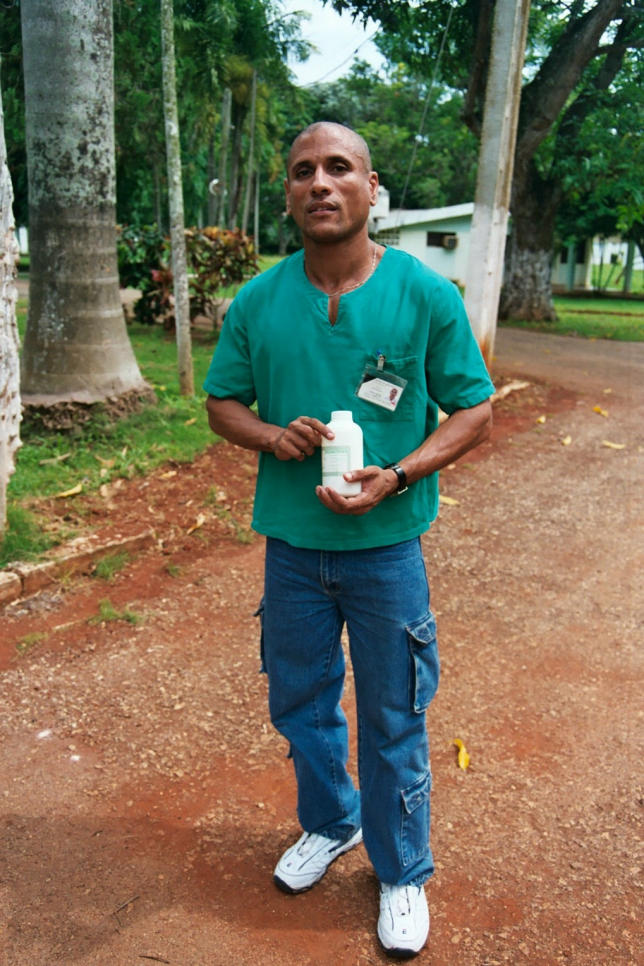
A physician in Havana, Cuba, holds a bottle of antiretroviral therapy (Photo: Arachu Castro)

However, suboptimal treatment adherence has been associated with virologic, immunologic, and clinical failure. In this essay, I look critically at the issue of adherence, and argue that, to address causes of incomplete adherence, we need to combine both quantitative and qualitative methodologies. These methodologies must be grounded in an understanding of adherence as a biological and social process that changes with time, and must be framed within an analysis of access to health care and medications.

## Adherence and Drug Resistance

A consensus exists that in order to achieve an undetectable viral load and prevent the development of drug resistance, a person on HAART needs to take at least 95% of the prescribed doses on time [1]. For many people, this means taking a regimen of three antiretrovirals twice per day—on both occasions, they are usually taking several pills [2]. An increasing number of studies show that the relationship between adherence and resistance is drug specific [3]. Although the suggestion that this relationship follows a bell-shaped curve has existed since just after the introduction of HAART [4], there is increasing evidence that drug resistance is highest among those taking 70%–80% of regimens containing a nonboosted PI (i.e., regimens with no combined ritonavir), and among those with intermittent or single-dose regimens of non-nucleoside reverse transcriptase inhibitors (including when nevirapine is used once to prevent mother-to-child transmission of HIV) or with poor adherence to these types of antiretrovirals. Ritonavir-boosted PIs (a full dose of a PI combined with ritonavir to increase the blood levels of the former) confer limited resistance, regardless of one's level of adherence [3].

Given the relevance of treatment adherence to improving life expectancy and preventing the spread of drug-resistant strains, many studies have attempted to predict causes of nonadherence in order to design strategies that reduce the number of missed doses. Except for some factors that have been associated with incomplete adherence in various settings, such as depression [5,6] or illegal drug use [7], study results are often inconclusive and do not yield comparable results—often due to conceptual and methodological differences among research protocols. Methodologically, there is growing agreement that patients' self-assessments of adherence—through interviews or self-administered questionnaires—show significant correlation with viral load tests [8–10], whereas estimations by their health-care providers often lead to invalid results [11].

Despite the current shortcomings in predicting who is more likely to miss doses of antiretroviral therapy (ART), the issue of adherence has become extremely important when setting priorities for allocating resources to fight AIDS in poor countries, where the majority of people who are HIV positive live. Some commentators have argued that adherence barriers are insurmountable in poor settings, so we should be cautious in delivering ART to these populations—a claim that is not grounded in evidence. In fact, adherence in poor settings is proving to be equal to, or even higher than, adherence in developed countries [12–17]. Furthermore, the argument about insurmountable adherence barriers in poor settings has also been challenged because it creates an unjustifiable double standard [11]—ART is not withheld from wealthy settings on the basis that many patients will skip doses.

## Adherence as a Biosocial and Dynamic Phenomenon

A biosocial approach to adherence relies on the dynamic analysis of the clinical and social course of disease and the continuous interaction of biological and social processes over time [18–21]. The study of pathology embedded in social experience captures a series of distal and proximal factors acting together—such as not seeking treatment for undesirable side effects due to lack of money to travel to a health center or purposely missing doses when one is asymptomatic to pretend that AIDS is not a concern. These factors shape the everyday life of patients, while patients internalize them and use them to provide meaning to their disease experience.

Years ago, before the advent of AIDS, anthropologists had noted that the introduction of effective therapy for a particular disease may profoundly alter the social interpretations of that disease [18,22]. Exposure to AIDS in Haiti, in the 1980s, generated cultural models of its etiology and expected course [19,23,24], which aimed to provide meaning to otherwise unknown phenomena—often locally interpreted in terms of jealousy and curse. Likewise, the social experience of AIDS in rural Haiti is also deeply affected by the advent of effective therapy, as preliminary data suggest that the introduction of quality HIV care can lead to a rapid reduction in stigma, with resulting increased uptake of testing [21].

Within the changing context in which disease may take place, adherence level is likely to change as biological and social circumstances, and interpretations of them, unfold. In Brazil, a boy and a girl living in a support house for children and adolescents orphaned by AIDS or living with HIV explained why, upon feeling well, they had started to delay the morning dose of antiretrovirals until the afternoon: “[The schedule] doesn't matter; you can take them any time, as long as you take them” [25]. Until they started HAART, they had both suffered several opportunistic infections and, at times, had been at the brink of death. Their incomplete adherence to the regimen was, in part, a lack of understanding about the importance of not missing doses—but, fundamentally, it was a strategy they had developed as an act of defiance against the rules of the support house, and probably to feel more like the children who were HIV negative who also lived there and were not taking daily medications [25]. By delaying the morning dose, they were providing their lives with a sense of normalcy, which had otherwise been characterized by orphanhood and chronic disease since early age, while acting out in protest.

By any account, these children would be classified as having incomplete adherence or, plainly, as nonadherent. Yet to improve their adherence, how helpful is it to know that they are not taking all their medications on time without understanding why? Could their nonadherence really be understood without analyzing the life trajectories of these children and their social context? Some studies approaching ART adherence within a dynamic framework have relied on a biosocial approach, providing a rich context in which taking medications occurs and evolves [7,9,26–28]. Other studies, while having observed that the level of adherence is not a static value, have remained mostly biomedical, examining the impact of side effects [29] or substance abuse [30], and have been devoid of the complexity of biosocial interactions and their changing nature, as in the case of the Brazilian children.

The study of life histories of patients—a standard qualitative method of ethnographic research—and of the interactions of social experience with illness episodes allows us to generate associations between the clinical and the social course of disease, including such themes as stigma, health-seeking behavior, or adherence to therapy. These associations, which are often drawn from a small sample of patients, can be validated by larger, statistically representative, epidemiological studies designed to include variables reflecting the social context of patients. For example, the effect of adipose tissue alterations (lipodystrophy)—a common side effect of PIs—on adherence to ART has often been studied without considering local patterns of ideal weight and body shape for women and men at different ages, and existing variations of these ideals related to the social position of patients. This lack of consideration for local patterns may explain why some studies arrive at opposite results—dystrophic weight gain as a barrier to or as an enhancer of adherence [29,31]. The effect of adherence on weight gain seen in patients on ART, as a result of a reversal of the disease process and general clinical improvements, should also be analyzed in relation to these social ideals. In Senegal, for example, where weight gain is a symbol of good health, such weight gain has been shown to increase adherence [26].

Most research studies on adherence to ART share the basic understanding that patients are adherent when they, after agreeing to the recommendations of a health-care provider [32], take the prescribed medications in a timely manner. However, an overemphasis on pill counting as a sum of discrete events limits our understanding of adherence as a complex process embedded in the clinical and social course of AIDS, as the case of the Brazilian children shows. An approach to adherence that combines both biological and social knowledge—a biosocial approach—and that relies on qualitative and quantitative methodologies is more likely to move us closer to a better understanding of adherence and, eventually, to improving adherence to ART.

## Poverty and Adherence

Partly because the introduction of ART in poor settings is recent, and partly because biomedical research rarely examines the social context in which patients live, there is a dearth of information on the direct effect of poverty on ART adherence. Most studies conducted in poor settings overlook how direct and indirect economic burdens borne by patients affect their ability to access a steady supply of antiretrovirals and take them on time. Such burdens may include the cost of missing work, the cost of elder or child care during medical visits, the cost of transportation to a health center, the cost of user fees, or the cost of tests and supplies. Although these costs may seem minimal to health professionals and decision makers, bearing these costs often translates into difficult household decisions about who eats, who works, or who goes to school. Taking medications in a timely manner may also require the challenging tasks of obtaining food and safe water, or of readjusting food intake to fit the drug-regimen schedule.

Despite the difficulties in overcoming these obstacles, the inability of a person living in poverty to obtain and take medications after initiating therapy is often labeled “noncompliance” or “nonadherence”—as has often occurred with tuberculosis patients [33,34]—and categorized as patient-related characteristics, ignoring social and economic causes or failures on the part of public-health interventions to address those causes [35]. Some studies conducted in Côte d'Ivoire [36], Senegal [28,37,38], and Botswana [39,40] show that user fees not only deter people from accessing AIDS care but also create an obstacle to treatment adherence. In other contexts where ART is free, such as Costa Rica, transportation costs have been associated with lower adherence [41]. The argument that patients would not value free drugs, or that free treatment might be humiliating for patients, are not borne out by higher adherence rates when drugs are free, such as in a comprehensive AIDS program in rural Haiti [14] or in Cuba [17], or when user fees are lowered, such as in Senegal [37,42]. Indeed, variations of directly observed therapy (DOT) for the delivery of ART (known as DOT-HAART) have proven useful in introducing complex multidrug regimens in poor settings lacking health-care infrastructures. In rural Haiti, for example, support for patients receiving DOT-HAART from community health workers improves rates of adherence [14].

As adequate and equitable access to comprehensive AIDS prevention and care are introduced, optimal adherence could be achieved if the multiple causes that shape patients' adherence are analyzed within their social context—including those related to the financing of health-care systems, and particularly cost-recovery mechanisms.

## A Biosocial Approach to Causes of Nonadherence: The Way Forward

The use of a biosocial framework grounded in the lived experience of people diagnosed with AIDS is essential to understanding adherence, the way adherence changes over time, and the reasons for nonadherence. Often times, particularly in poor settings, these reasons will be found outside the individual responsibility of patients. Addressing adherence may require providing social support to patients, lowering or eliminating user fees, bringing health-care workers closer to patients, opening health centers focused on patients' competing demands to survive, improving drug procurement strategies, or creating mechanisms for lowering the cost of drugs and lab tests. In many cases it will mean improving and investing in primary health care, public hospitals, and referral networks, or in incentives to recruit and retain health-care workers committed to serving their patients. Given this complexity, the possibility of advancing the understanding of the multifaceted causes of nonadherence needs to be analyzed within its larger social, economic, and political context.

Box 1 shows the main biosocial variables needed to analyze adherence to ART defined within eight broad categories: socioeconomic factors, health-care system, social capital, cultural models of health and disease, personal characteristics, psychological factors, clinical factors, and antiretroviral regimen. Most clinical studies have focused on the last four areas, which are easier to measure quantitatively but which do not account for the larger social context. Although clinical epidemiological studies are essential to finding associations between drug regimens and adherence—and, depending on the method chosen, to establishing causality—a biosocial approach that combines quantitative and qualitative methodologies is necessary to bridge the current gap in knowledge on adherence to ART. Only by understanding the complicated interplay between the clinical and social factors that affect adherence to ART can we hope to overcome the real causes of nonadherence.

**Box 1. Biosocial Variables for Understanding Adherence to ART**

**Socioeconomic Factors**

- Poverty and inequality
- War, political violence
- Cost of medications
- Cost of CD4+ counts, viral load
- Transportation costs
- Cost of missing days from work
- Cost of food and safe water
- Costs associated to changes in lifestyle
- Other costs

**Health-Care System**

- Health-care infrastructure
- Drug stock shortages
- Financing mechanisms (including user fees)
- Quality of relationship with health-care providers

**Social Capital**

- Kinship patterns
- Networks of social support
- Social status
- Homelessness, incarceration

**Cultural Models of Health and Disease**

- On etiology and transmission
- On health-care providers and healers
- On cure
- On efficacy and toxicity of drugs
- On sick role

**Personal Characteristics**

- Age
- Sex and gender
- Ethnic group
- Education
- Occupation
- Household composition
- Substance abuse
- Physical disabilities

**Psychological Factors**

- Self-esteem and motivation
- Mental health conditions

**Clinical Factors**

- Immunological or clinical stage of HIV disease
- Occurrence and severity of opportunistic infections
- Side effects (desirable and undesirable)
- Symptomatology at onset of treatment
- Effect of pregnancy or lactation

**Antiretroviral Regimen**

- Number of drug regimens per day
- Number of pills per regimen
- Therapeutic class composition of drug regimen

## Abbreviations

ART: antiretroviral therapy
DOT: directly observed therapy
HAART: highly active antiretroviral therapy
PI: protease inhibitor
